# Metformin increases bone marrow adipose tissue by promoting mesenchymal stromal cells apoptosis

**DOI:** 10.18632/aging.204486

**Published:** 2023-01-14

**Authors:** Wu Duan, Huajie Zou, Nan Zang, Dongxia Ma, Bo Yang, Lin Zhu

**Affiliations:** 1 Department of Endocrinology, Qilu Hospital of Shandong University, Jinan, Shandong 250012, China; 2 Department of Endocrinology, The Affiliated Hospital of Qinghai University, Xining 810000, China; 3 Department of Allergy, Tongji Hospital, Tongji Medical College, Huazhong University of Science and Technology, Wuhan 430030, China; 4 Institute of Organ Transplantation, Tongji Hospital, Tongji Medical College, Huazhong University of Science and Technology, Wuhan 430030, China; 5 Department of Pediatrics, Tongji Hospital, Tongji Medical College, Huazhong University of Science and Technology, Wuhan 430030, China

**Keywords:** bone marrow mesenchymal stem cells (BM-MSCs), bone marrow adipose tissue (MAT), adipogenesis, osteogenesis, apoptosis

## Abstract

Bone marrow adipose tissue (MAT) has the potential to exert both local and systemic effects on metabolic homeostasis. As a first-line drug used to treat type 2 diabetes mellitus, metformin has conflicting effects on MAT and bone marrow mesenchymal stem cell (BM-MSC) differentiation. Through a series of experiments *in vivo* and *in vitro*, we found that except improving the glucose and lipid metabolism disorder in *ob*/*ob* mice, 200 mg/kg metformin increased MAT in mice tibia, and prompted osteogenic genes (*RunX2, OPN, OCN*) and lipogenic genes (*Ppar-γ, Cebpα, Scd1*) expression in mice bone marrow. However, metformin promoted osteogenesis and inhibited lipogenesis of MSC *in vitro*, which is inconsistent with the results *in vivo*. Given MAT being considered the “filler” of the space after the apoptosis of bone marrow stroma, the effect of metformin on MSC apoptosis was examined. We discovered that metformin induces MSC apoptosis *in vivo* and *in vitro*. Therefore, we speculated that the increased MAT in mice tibia may be attributed to the filling of adipose tissue after apoptosis of bone marrow stromal cells induced by metformin. The increased MAT may be involved in the regulation of metformin on glucose, lipid, and bone metabolism in diabetic mice, providing a new way to understand the metabolic regulation of metformin. While increased MAT-associated insulin resistance and metabolic disorders may account for the poorer clinical benefits in patients with intensive glucose control.

## INTRODUCTION

Bone marrow adipose tissue (MAT) is considered the “filler” of the space after bone marrow senescence and bone marrow stromal cell apoptosis [1]. As an active endocrine organ, MAT regulates the secretion of active molecules such as leptin, adiponectin, IL-6, and TNF-α [2, 3]. MAT, was associated with aging, obesity, diabetes, and osteoporosis [4, 5]. Bone marrow mesenchymal stem cells (BM-MSCs) are non-hematopoietic stem cells capable of differentiating into osteoblasts, chondroblasts, and adipocytes [6]. The ratio of osteoblasts and adipocytes determined the content of adipose tissue in bone marrow [7].

Multiple factors, including chemical, physical and biological factors, such as bone injury, anorexia, irradiation therapy, thiazolidinediones, and glucocorticoids [4], regulate the balance of adipogenic and osteoblastic differentiation of MSCs [7]. MAT was involved in metabolic regulation in patients with diabetes [8], but excessive bone marrow adipose tissue was associated with metabolic disorders, and increased risk of osteoporosis and bone fracture [7, 9].

As a first-line hypoglycemic treatment for type 2 diabetes mellitus (T2DM) patients, metformin has been proven in several major studies for its hypoglycemic, weight-reducing and anti-inflammatory effects [10]. However, its effect on bone marrow adipogenesis in diabetic patients and its related mechanisms have not been fully elucidated. Metformin has conflicting effects on bone marrow adipogenesis or differentiation of mesenchymal stem cells. Studies found that metformin promoted the differentiation of MSC into osteoblasts but not adipocytes by activating osteogenic genes [runt-related transcription factor 2 (*RunX2*), bone sialo protein (*Bsp*), osteopontin (*OPN*)] and inhibiting lipogenic genes [peroxisome proliferator-activated receptors (*PPAR*), CAAT/enhancer binding proteins (*C/EBP*), *aP2* (a fatty acid binding protein)] through inducing endothelial nitric oxide synthase (*eNOS*) expression [11], activating Adenosine 5′-monophosphate-activated protein kinase (*AMPK*) signaling pathway [12] or down-regulating the expression of glycogen synthase kinase 3 beta (*GSK3β*) [13]. Moreover, metformin reduces MAT and increases bone formation, reducing the risk of fracture in obese mice [14]. However, other studies illuminated that, metformin, an AMPK activator, did not promote the osteogenic differentiation of human amnion-derived MSCs (hAMSCs) and MSCs in both growth medium and osteogenic medium [15], inhibited gene expression of *Runx2* and osteoblast differentiation markers including osteocalcin (*Ocn*), *Bsp*, and *Opn* in primary osteoblasts and MC3T3-E1 cells (a mouse osteoblastic cell line) [16]. Those conflicting studies have confused our understanding of the role of metformin in MAT.

To clarify the effect of metformin on MAT while lowering blood glucose, *ob/ob* mice, a classic T2DM model, were used as the research object to investigate the effect of metformin on bone marrow adipogenesis, as well as on adipogenic and osteogenic differentiation of primary BM-MSC, and to explore the related mechanisms.

## MATERIALS AND METHODS

### Animals and treatment

*Ob/ob* mice and C57BL/6J mice, female, 6 weeks old, were purchased from Beijing HFK Bioscience Co., Ltd. (Beijing, China). After 1 week of acclimatization, the groups of C57BL/6J and *ob/ob* mice were further split into two subgroups (*n* = 6) randomly that were treated with 200 mg/kg·d of metformin (Merck KGaA, SigmaAldrich, St. Quentin-Fallavier, France) or placebo for 16 weeks. Metformin was provided in the drinking water during the 16 weeks. The quantity of metformin added to the drinking water was calculated based on daily liquid consumption in both groups (~5–6 and 8–9 mL per mouse in C57BL/6J and *ob/ob* mice, respectively). Body weight and random blood glucose were evaluated regularly. At the end of the treatment, ITT was performed and then mice were sacrificed for the sampling of blood, liver, and tibia. The liver, and tibia was rapidly processed for appropriate histological staining and TUNEL staining. The serum was subsequently stored at −80°C for the subsequent biochemical tests.

All procedures used in this study involving animals were performed and monitored following the guidelines of the Chinese Council on Animal Care and were approved by the Institutional Animal Care and Use Committee of the Tongji Medical College, Huazhong University of Science and Technology. Throughout the study, all efforts were made to minimize the suffering of the animals.

### Biochemical analysis

Blood lipid (triglyceride (TG), total cholesterol (TC), high-density lipoprotein cholesterol (HDL-C), low-density lipoprotein cholesterol (LDL-C)) and insulin levels were measured using a standard clinical automatic analyzer in the clinical laboratory of Tongji Hospital, Tongji Medical College, Huazhong University of Science and Technology.

### Pathological analysis

Mice’s liver and tibia were fixed in 10% neutral-buffered formalin solution. Tibia was decalcified in EDTA decalcified fluid (G1105, Servicebio), followed by paraffin embedding. Then, the livers and tibia were cut into 5 μm-thick sections and stained with hematoxylin and eosin (HE). The degree of lipid infiltration on liver HE staining was scored on a scale of 0–4 with 0 being normal healthy tissue and 4 being the worst, as described previously [17]. Marrow adipocyte area and number quantification were analyzed according to Styner and colleagues [18].

### TUNEL staining

For the detection of apoptosis, paraffin-embedded tibia sections were stained with the TUNEL technique using an *in-situ* cell death detection kit (Roche, Basel, Switzerland) according to the manufacturer’s protocols. DAPI staining for nucleus. Under the fluorescence microscope (Olympus, Tokyo, Japan), the TUNEL-positive cells exhibited green fluorescence. The apoptosis index was calculated as the percent of TUNEL-positive cells relative to the total number of cells using ImageJ software.

### qRT-PCR

Total RNA was isolated from the whole tibia marrow. Osteogenic genes (*RunX2, OPN, OCN*), adipogenic genes [peroxisome proliferator-activated receptor-γ (*Ppar-γ*), CCAAT/enhancer-binding protein-α (*Cebpα*), Stearyl CoA desaturase 1(*Scd1*)] and apoptosis-related genes (Bcl-2-associated X protein (*Bax*), B-cell lymphoma-2 (*Bcl-2*), Bcl-2-associated agonist of cell death (*Bad*)) mRNA expression in tibia bone marrow of C57BL/6 and *ob/ob* mice were analyzed by quantitative reverse transcription-polymerase chain reaction (qRT-PCR). Using a StepOnePlus Real-Time PCR System to evaluate gene expression, the relative gene expression was estimated using the 2−ΔΔCT technique with *GAPDH* serving as the reference gene. The primer sequences used for the experiment are exhibited in Table 1. All the primers were designed and synthesized in TsingKe Biotechnology Co., Ltd, Wuhan.

**Table 1:** Specific primers used for RT-PCR.

**Gene**	**Primer sequences**	
	**Forward**	**Reverse**
*RunX2*	CTGCCACCTCTGACTTCTGC	GATGAAATGCCTGGGAACTG
*OPN*	TTCTCCTGGCTGAATTCTGAGG	GCTATAGGATCTGGGTGCAGG
*OCN*	CTTGGTGCACACCTAGCAGA	GCCGGAGTCTGTTCACTACC
*Ppar-γ*	CCACAGTTGATTTCTCCAGCAT	TCCCCACAGACTCGGCAC
*Cebp-α*	GTGGACAAGAACAGCAACGAG	ACGTTGCGTTGTTTGGCTTTA
*Scd1*	AGTTCCGCCACTCGCCTACA	GGCACCGTCTTCACCTTCTC
*Bax*	AGACAGGGGCCTTTTTGCTAC	AATTCGCCGGAGACACTCG
*Bcl-2*	GCTACCGTCGTGACTTCGC	CCCCACCGAACTCAAAGAAGG
*Bad*	CAGCCACCAACAGTCATCAT	CCTCAAACTCATCGCTCATC
*GAPDH*	GACAAAATGGTGAAGGTCGGT	GAGGTCAATGAAGGGGTCG

### Isolation, culture, and treatment of BM-MSCs

BM-MSCs were obtained from C57BL/6 mice and cultured as previously described [19, 20]. The identification of primary BM-MSCs was confirmed by morphology, phenotypic analysis, and their ability to differentiate into adipocytes and osteoblasts [19, 20]. To choose the appropriate concentration of metformin, BM-MSCs were treated with 0, 1, 5, and 10 mM/L metformin, then cell viability was determined by CCK-8 assay and cell apoptosis was analyzed using flow cytometric analysis. To explore the effect of metformin on adipogenic and osteogenic differentiation of primary BM-MSCs, cells were treated with 1 mM/L metformin during the differentiation process. Oil red O and Alizarin Red S staining were performed to identify the oil droplets and calcification in the differentiated cells respectively.

### Cell viability assay

A CCK-8 kit was used to measure cell viability (Dojindo, Kumamoto, Japan). 5000 cells/well of cells were seeded in 96-well plates, and the cells were then incubated for 24 hours. Following metformin treatment (0, 1, 5, and 10 mM/L) as previously mentioned, 10 μL of the CCK-8 solution was added to each well, and the cells were cultured for an additional 100 minutes at 37°C. Finally, using a microplate reader, the absorbance values were calculated at 450 nm.

### Flow cytometric analysis

Cell apoptosis was analyzed with an Annexin V-FITC and propidium iodide (PI) staining kit according to the manufacturer’s instruction (MultiSciences, Hangzhou, China). In brief, BM-MSC were digested and centrifuged at 300 × g for 5 minutes, and then incubated for 5 minutes in the dark with Annexin V-FITC and PI. The percentages of apoptotic cells were measured using flow cytometry on a FACSCalibur (BD Biosciences, NJ, USA). Data analysis was done with the help of FlowJo software (PR, USA).

### Statistical analysis

All data are represented as mean ± standard error of the mean (S.E.M). Significant differences between the two groups were performed by two-tailed Student’s *t*-test for independent variables. Differences among groups were evaluated by one-way ANOVA followed by posthoc testing or MNOVA. A value of *P* < 0.05 was considered statistically significant.

### Availability of data and materials

The raw data of the study can be provided upon request with maintenance of confidentiality, privacy, and anonymity of the research participants.

## RESULTS

### Metformin reduced body weight and alleviated glucose and lipid metabolism disorders in *ob/ob* mice

**Figure 1:**
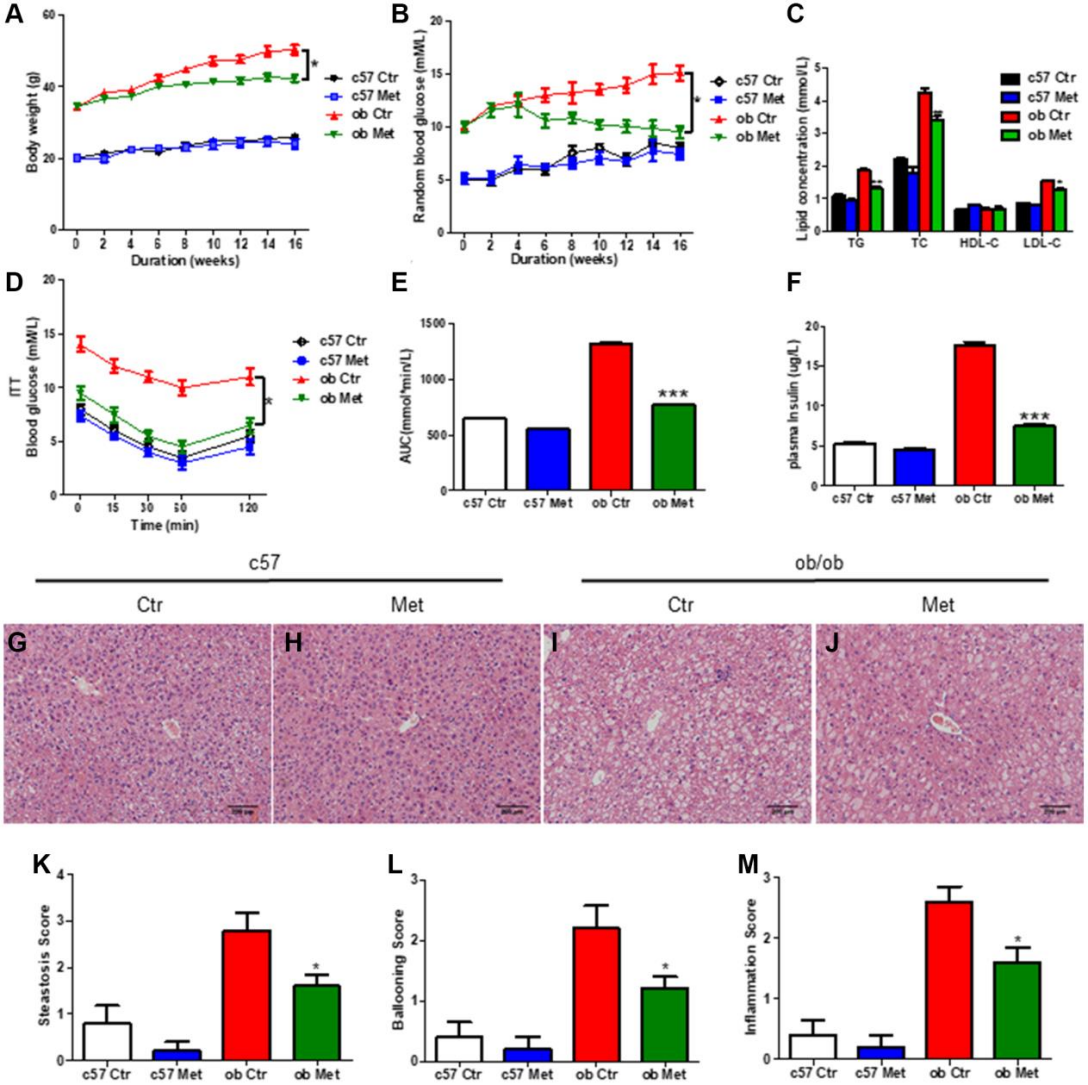
**Metformin reduced body weight and alleviated glucose and lipid metabolism disorders in *ob/ob* mice.** C57BL/6 and *ob/ob* mice were treated with 200 mg/kg metformin for 16 weeks. Body weight (**A**) and random blood glucose (**B**) were presented every 2 weeks. The level of serum TG/TC/LDL-C and insulin were exhibited in (**C** and **F**) respectively. (**D**, **E**) The curve and area under the curve (AUC) of ITT in each group. Liver sections were prepared from C57BL/6 and *ob/ob* mice, HE (**G**–**J**), and evaluation of hepatic steatosis (**K**), ballooning (**L**) and inflammation (**M**) was carried out. Scale bar = 200 μm. Data are presented as mean ± S.E.M. ^*^P < 0.05, ^**^P < 0.01, ^***^P < 0.001 vs. ob ctr.

As shown in Figure 1A, metformin significantly reduced body weight in *ob/ob* mice but not in C57BL/6 mice (*P* < 0.05). The level of TG, TC, and LDL-C (Figure 1C), random blood glucose level in the experimental process (Figure 1B), and AUC of ITT (Figure 1D, 1E) of *ob/ob* mice treated with 200 mg/kg metformin were lower than control *ob/ob* mice. Moreover, metformin decreased the plasma insulin level in *ob/ob* mice (Figure 1F), indicated metformin alleviated the insulin resistance in *ob/ob* mice.

Hepatic HE staining revealed significant lipid deposition (Figure 1I) in *ob/ob* mice, and increased hepatic steatosis (Figure 1K), hepatocyte ballooning (Figure 1L), and intralobular inflammation scores (Figure 1M) were obtained, all of which were substantially lessened in the metformin-treated mice livers. (Figure 1J), but not in C57BL/6 mice (Figure 1G, 1H). Taken together, metformin distinctly alleviated glucose and lipid metabolism disorder in diabetic *ob/ob* mice.

### Metformin increased MAT in normal and diabetic mice

**Figure 2:**
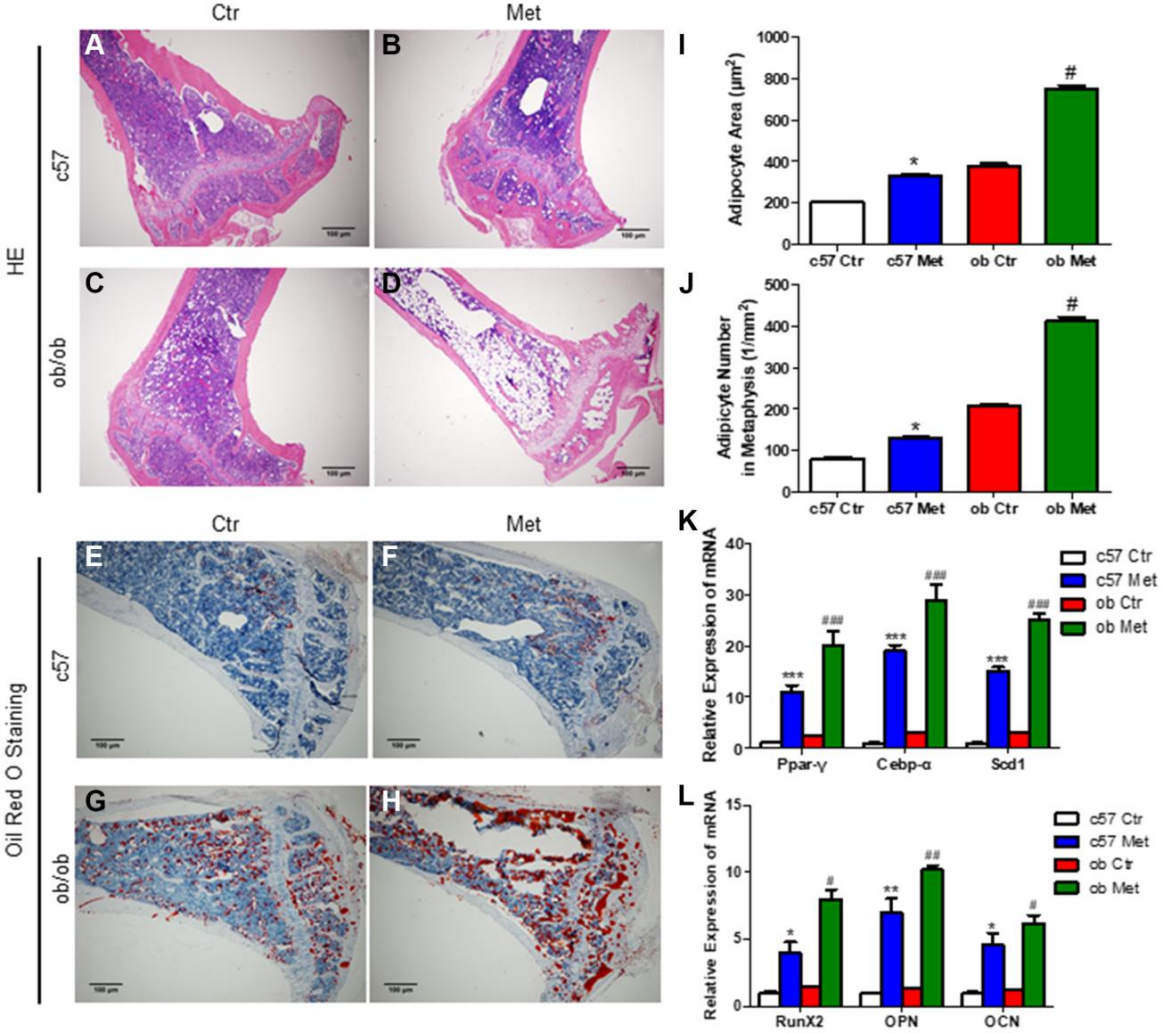
**Metformin increased MAT in C57BL/6 and *ob/ob* mice.** Adipocyte size was assessed in high-power field images of the proximal tibia. Representative images of HE staining (**A**–**D**) and oil red O staining (**E**–**H**) for each group. (**I**) Area of adipocytes represented as mean ± SEM. (**J**) The number of adipocytes per square millimeter. mRNA relative expression of adipogenic genes (*Ppar-γ*, *Cebp-α*, *Scd1*) (**K**) and osteogenic genes (*RunX2, OPN, OCN*) (**L**) in the bone marrow. Data are presented as mean ± S.E.M. ^*^P < 0.05, ^**^P < 0.01, ^***^P < 0.001 vs. c57 ctr. ^#^*P* < 0.05, ^##^*P* < 0.01, ^###^*P* < 0.001 vs. ob ctr.

Interestingly and surprisingly, our HE and Oil red O staining of the proximal tibia revealed an increase in the number of adipocytes in the metformin-treated group compared with Ctr group (Figure 2A, 2C and 2E, 2G), this effect was more pronounced in *ob/ob* mice (+97%, Figure 2D, 2H, 2J) than in C57BL/6 mice (+63%, Figure 2B, 2F, 2J). Meanwhile, we determined the average size of clearly demarcated adipocytes within HE-stained histologic sections using Image J according to Styner’s methods [18], which shows that bone marrow adipocytes in metformin-treated groups were larger than those in the control group. And metformin increased the average size of each adipocyte irrespective, by 63% in C57BL/6 and 97% in *ob*/*ob* mice (Figure 2I).

We also analyzed the changes of adipogenic and osteogenic genes in tibia bone marrow of C57BL/6 and *ob/ob* mice, as shown in Figure 2K, 2L, metformin increased the mRNA level of *Ppar-γ* (a regulatory factor of lipid transport and storage), *Cebp-α* and *Scd1* (a key regulatory gene for monounsaturated fatty acid synthesis) distinctly, which were important adipogenic transcription factors. While, the mRNA level of osteogenic genes *RunX2*, *OPN* and *OCN* were also increased in the metformin-treated group, both in C57BL/6 and *ob/ob* mice.

Those results indicated that metformin increased MAT in the tibia both in C57BL/6 and *ob/ob* mice. As we all know that metformin, as an activator of AMPK, promotes osteogenesis and inhibits lipogenesis by regulating the expression of genes related to osteogenesis and lipogenesis in a variety of cell and animal models [13, 21]. To further clarify the effect of metformin on osteogenic and adipogenic differentiation and explore its mechanism of increasing bone marrow adipogenesis in C57BL/6 and *ob/ob* mice, we examined the effect of metformin on MSCs apoptosis, adipogenesis, and osteogenesis *in vitro*.

### Metformin promoted MSC apoptosis in a concentration-dependent manner and facilitated osteogenesis and inhibited adipogenesis in MSC

**Figure 3:**
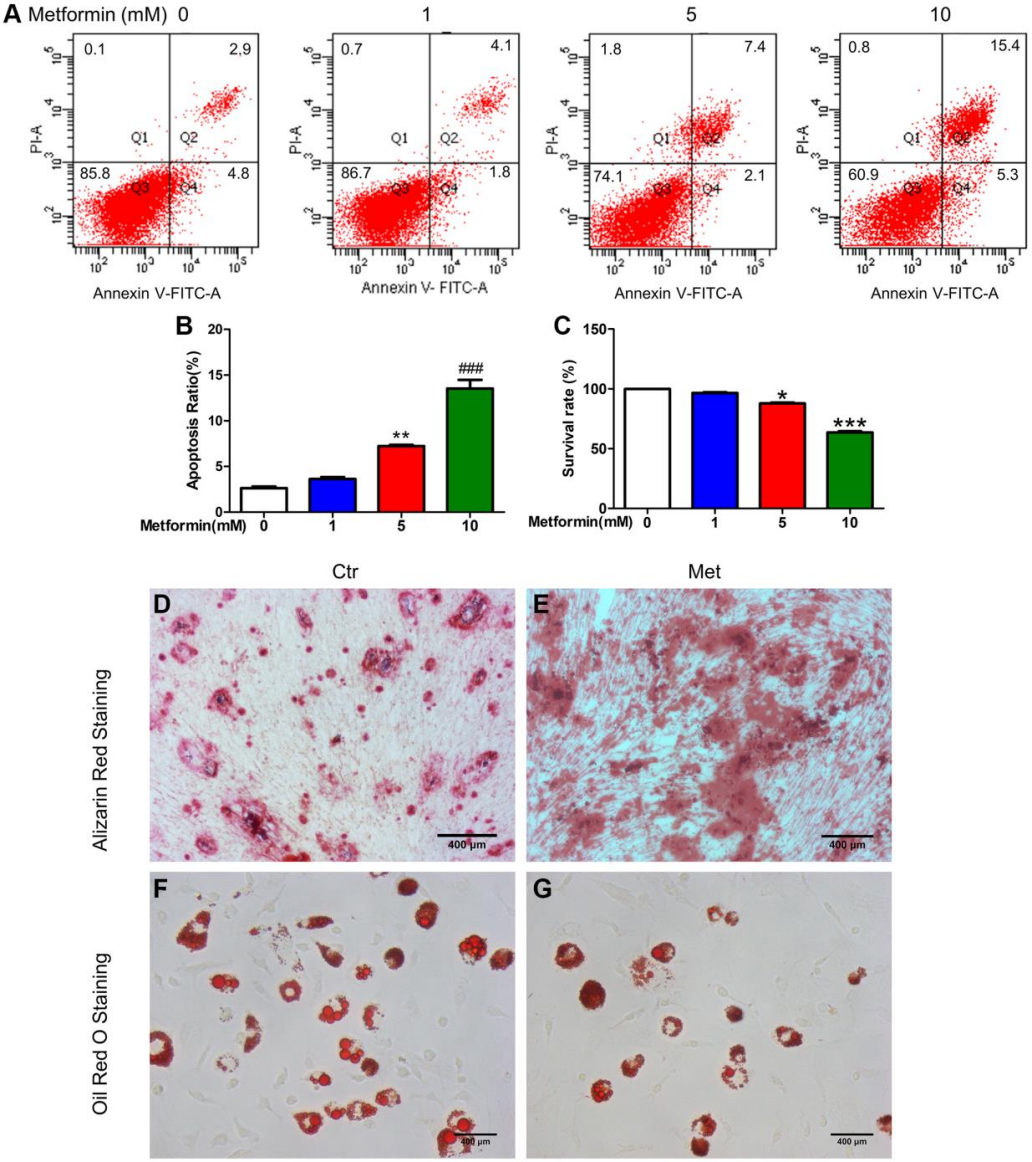
**Metformin promoted MSC apoptosis in a concentration-dependent manner, promotes BM-MSC osteogenesis, and inhibits adipogenesis *in vitro*.** BM-MSCs were treated with 0, 1, 5, and 10 mM/L metformin, then cell viability was determined by CCK-8 assay and cell apoptosis was analyzed using flow cytometric analysis. (**A**) Flow apoptosis detection induced by different concentrations of metformin in MSC. (**B**) Statistics of the apoptosis rate for each group. (**C**) Cell activity assay about the effects of different concentrations of metformin on MSC. Cell Counting Kit-8 was used for the cell activity assay. BM-MSCs were treated with 1 mM/L metformin during the adipogenic and osteogenic differentiation process. Alizarin Red (**D**, **E**) and Oil red O staining (**F**, **G**) for each group cell.

To select the appropriate concentration of metformin, we treated MSCs with 0, 1, 5, and 10 mM metformin. As shown in Figure 3A–3C, 1 mM metformin do not affect MSCs viability and apoptosis, with the increasing of concentration, 5 mM and 10 mM metformin impaired MSCs viability and induced apoptosis, and those effects were more obvious in the 10 mM group. So, 1 mM metformin was used in the subsequent experiments. Oil red O and Alizarin Red S staining in Figure 3D–3G indicated that 1 mM metformin promoted MSCs differentiated into osteoblasts while inhibiting adipogenesis.

### Metformin promoted marrow stromal cell apoptosis in *ob/ob* mice

**Figure 4:**
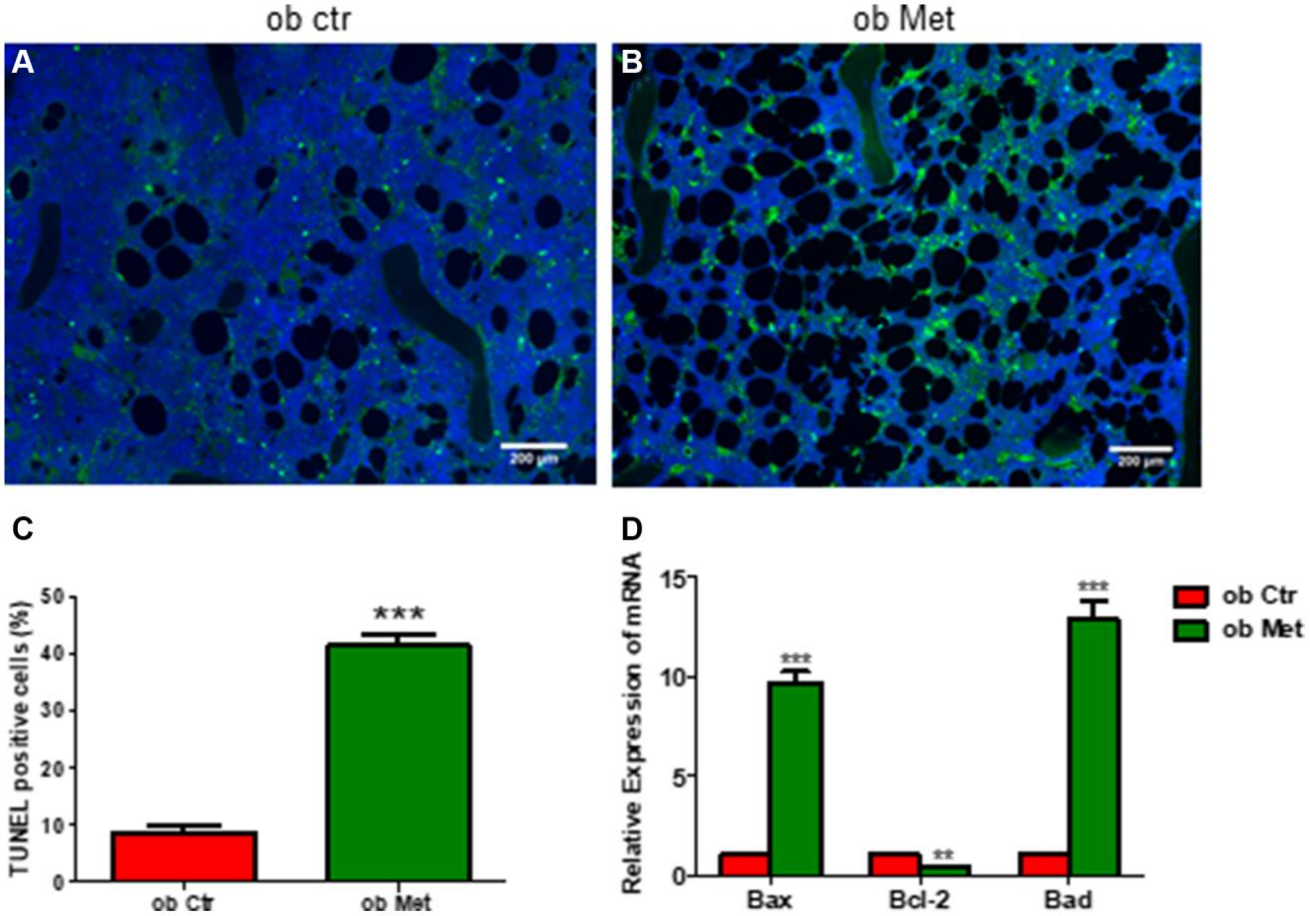
**Metformin increased bone marrow cell apoptosis in *ob/ob* mice.** (**A**, **B**) TUNEL staining in control and metformin-treated mice tibia sections (scale bar = 200 mm). (**C**) Quantification of TUNEL-positive cells in the proximal tibia. (**D**) mRNA relative expression of apoptosis-related genes (*Bax*, *Bcl-2*, *Bad*) in the bone marrow. Data are presented as mean ± S.E.M. ^**^P < 0.01, ^***^P < 0.001 vs. ob ctr.

Interestingly, TUNEL staining in Figure 4 of the tibia showed that the percentage of TUNEL-positive cells was higher in metformin-treated *ob/ob* mice (Figure 4B, 4C) compared with Ctr *ob/ob* mice (Figure 4A). Moreover, metformin increased the mRNA level of apoptosis-related genes, including *Bax*, *Bcl-2*, and *Bad*, indicating that 200mg/kg metformin induces apoptosis of marrow stroma cells in *ob/ob* mice (Figure 4D).

## DISCUSSION

Our study showed that metformin alleviated the glucose and lipid metabolism disorders in diabetic *ob/ob* mice, and increased the MAT of the tibia in C57BL/6 and *ob/ob* mice, while promoting osteogenesis and inhibiting lipogenesis in MSC. To investigate the mechanism of this contradictory effect of metformin *in vivo* and *in vitro*, we explored the effect of metformin on the apoptosis of MSCs. We found that a high concentration of metformin (5 and 10 mM) induced apoptosis of MSC *in vitro*, and 200 mg/kg metformin increased apoptosis of bone marrow stromal cells in *ob/ob* mice. We speculated that the increased MAT in *ob/ob* mice may be attributed to the filling of adipose tissue in mice tibia after metformin-induced apoptosis of bone marrow stromal cells.

Numerous studies have demonstrated the varying influences on MAT. After radiation^+/−^ chemotherapy for bone marrow transplantation, marrow adipogenesis is florid, and the adipocytes fill the bone marrow space throughout the mouse skeleton [22]. Furthermore, aging, persistent unloading (such as in patients with paraplegia or those on prolonged bed rest) or intermittent exposure to reduced forces (such as in astronauts exposed to microgravity) [23], and obesity but also caloric restriction and anorexia, thiazolidinediones (PPAR-γ inhibitor), and glucocorticoids, promote MAT accumulation [4]. With aging, decreasing levels of stromal-derived factor (SDF-1), insulin-like growth factor (IGF-1), and side population (SP) stem cells may be linked to increased MAT [24]. Given the known hormonal abnormalities in anorexia nervosa patients, early hematopoietic to adipogenic transition may expedite adipocyte conversion over osteoblast differentiation in the mesenchymal stem cell pool, leading to increased bone marrow adipogenesis and early conversion to yellow marrow [25]. Whereas in type 1 diabetes mellitus (T1DM) patients, poor metabolic control alters the GH/IGF-1 (growth hormone/Insulin-like growth factors-1) axis, whereas higher urine magnesium excretion may signal modest changes in renal function and/or glucosuria that result in smaller and less dense bones [26]. But few studies have focused on changes in MAT, and studies of the mechanisms involved have not been reported.

Additionally, MAT is regarded as the “filler” of the spaces following bone marrow stromal apoptosis [1]. He et al. found that metformin-induced mouse mesenchymal stromal cell apoptosis through AMPK-mediated mTOR suppression, which dampened its cardioprotective effect after transplantation into infarcted hearts in diabetes [27]. They also discovered that under intense glucose control in *db*/*db* diabetic mice, metformin triggered MSC apoptosis by the same signaling mechanism, which may help to explain the lower therapeutic benefit of the intensive glucose control strategy [28]. Another study reported that metformin-induced apoptosis in epithelial ovarian cancer by activating caspases 3/7 activity, decreasing Bcl-2 and Bcl-xl expression, and increasing Bax and Bad expression [29]. In line with this study, we discovered that metformin increased the expression of Bax and Bad mRNA while decreased the expression of Bcl-2 mRNA, leading to MSC apoptosis. The increased MAT of the tibia may be attributed to the filling of adipose tissue after metformin-induced apoptosis of bone marrow stromal cells in *ob/ob* mice in our study, the exact mechanism has not been elucidated, we speculate that this may be a form of self-repair of the bone marrow tissue.

Preclinical investigations have revealed that metformin promotes osteogenic differentiation and inhibits lipogenic differentiation of primary bone marrow stromal stem cells, inhibit adipogenesis of 3T3-L1 cells through the AMPK-Gfi1-OPN axis, also has osteogenic effects on bone marrow progenitor cells [30], promotes osteoblast activity and reduces osteoclastogenesis [31], possibly through activation of the AMPK and subsequently Runx2 [32], and attenuates the inhibitory effects of hyperglycemia on osteoblast activity [33]. However, clinical studies have reported that metformin reduces or has no effect on fracture risk [34]. Intriguingly, our study demonstrated that metformin has different effects on MSC about adipogenic *in vivo* and *in vitro*. According to pharmacogenetic studies, plasma metformin concentrations vary considerably between individuals [35]; this may explain the divergence in findings in the past. The effects of metformin on the MAT and bone matrix in T2DM patients need to be further elucidated.

In conclusion, we found that apart from improving the glucose and lipid metabolism disorder in obese diabetic mice, 200 mg/kg metformin increased MAT in *ob*/*ob* mice, which may be attributed to metformin-induced apoptosis of bone marrow stromal cells. *In vitro*, metformin promoted osteogenesis and inhibited lipogenesis of MSC, and induced MSC apoptosis in a concentration-dependent manner. The seemingly contradictory effects of metformin on the lipogenesis of bone marrow stromal cells *in vivo* and *in vitro* may be involved in its pro-apoptosis effect on bone marrow stromal cells.

In addition to serving as a major “endocrine organ,” MAT has the potential to affect metabolic homeostasis, skeletal remodeling, hematopoiesis, and bone metastasis development on both a local and a systemic level [3]. In diabetic mice, increased MAT may regulate glucose, lipid, and bone metabolism, providing new insights into metformin metabolism. MSC’s cardiac protective properties are weakened by metformin-induced apoptosis, which may explain why patients with more intensive glucose control experience fewer clinical benefits. Metformin-induced increased MAT, which leads to insulin resistance and metabolic disorders in obese/obese mice [3], may account for the poorer clinical benefits in patients with intensive glucose control. Due to these reasons, metformin should be considered in clinical applications based on its effects on differentiation, proliferation, and apoptosis in BM-MSCs, as well as its increased risk associated with MAT. It remains to be determined whether increased MAT and changes in bone mineral density increase fracture risks in diabetic mouse despite metformin increasing osteogenic-related gene expression. Further research is needed to determine whether metformin increases fracture risk in diabetic patients through similar mechanisms.
